# Synergistic effect of graphene oxide and zoledronic acid for osteoporosis and cancer treatment

**DOI:** 10.1038/s41598-020-64760-4

**Published:** 2020-05-08

**Authors:** Gökçen Boran, Sepideh Tavakoli, Ingo Dierking, Ali Reza Kamali, Duygu Ege

**Affiliations:** 1 Boğaziçi University, Institute of Biomedical Engineering, İstanbul, Turkey 34368; 2 Northeastern University, College of Engineering, Boston, Massachusetts USA 02115, Boğaziçi University, Institute of Biomedical Engineering, İstanbul, Turkey 34368; 3 University of Manchester, Department of Physics and Astronomy, Manchester, M13 9PL United Kingdom; 4 Energy and Environmental Materials Research Centre (E2MC), School of Metallurgy, Northeastern University, Shenyang, China 110819

**Keywords:** Biomaterials, Biomaterials

## Abstract

Zoledronic acid (ZOL) is a third generation bisphosphonate which can be used as a drug for the treatment of osteoporosis and metastasis. In this study, graphene oxide (GO) is conjugated with ZOL, and the nanostructured material is evaluated in terms viability, proliferation and differentiation. Furthermore, the associated morphological changes of bone marrow-derived mesenchymal stem cells (BM-MSC), and Michigan Cancer Foundation-7 (MCF-7) breast cancer cells, as well as the effect of the drugs on mineralization of BM-MSCs are investigated using a variety of characterization techniques including Fourier Transform Infrared Spectroscopy (FTIR), scanning electron microscopy (SEM) as well as alamar blue, acridine orange, and alizarin red assays. Nanostructured ZOL-GO with an optimum performance is synthesized using ZOL and GO suspensions with the concentration of 50 µM and 2.91 ng/ml, respectively. ZOL-GO nanostructures can facilitate the mineralization of BM-MSC cells, demonstrated by the formation of clusters around the cells. The results obtained confirm the performance of ZOL-GO nanostructures as promising drug complexes for the treatment of osteoporosis and metastasis.

## Introduction

Secondary bone cancer (metastasis) occurs when cancer spreads to the bones from different organs including lung, kidney, thyroid^1–3^, and particularly from the prostate and breast, as observed in 70% of cases^2^. Bisphosphonates have commonly been used for decades as a treatment for metastasis^4,5^. Zoledronic acid (ZOL) is a more recently developed bisphosphonate which is found to be more potent in the treatment of metastasis^6–11^. Despite this, the utilization of small ZOL molecules in this application is difficult, since the free drug can easily be filtrated before arriving at the tumor site^12,13^. As a result, higher doses of the drug must be administered which increases the chance for the occurrence of side effects, including acute systemic inflammatory reactions, osteonecrosis of the jaw, renal failure, nephrotic syndrome, electrolyte imbalance and ocular inflammation^14–16^. To increase the efficiency of the drug and reducing the chance of side effects, ZOL can be loaded on different drug carriers such as poly(lactide-co-glycolide)^17^, folate targeted liposomes^18^, β-tricalcium phosphate^19^, hydroxyapatite^20^ and gelatin^21^.

Carbon allotropes show potential as alternatives to conventional drug carriers due to their high surface area and biocompatibility as well as the capability of being chemically modified^22^. In particular, graphene oxide (GO), carbon nanotubes (CNT), nanodiamond and carbon black are known to be capable of delivering drugs into cancer cells, improving the efficiency of the drug^23^. Among these carbons, GO has obvious advantages such as low cost, the presence of two external chemically active surfaces, easy fabrication and modification, and the absence of toxic metal particles during its production^24^. Studies also indicated that the loading capacity of GO is higher than CNT and carbon black^25,26^. Moreover, the degree of cytotoxicity of CNT and carbon black are found to be higher than GO^27^. Therefore, in this study, GO was chosen as the preferred carrier for ZOL.

GO is the oxidized form of graphene, functionalized with groups such as hydroxyls, epoxides, diols, ketones, and carboxyls^28^. GO has been used as a drug carrier for anti-cancer drugs such as doxorubicin^29^, camptothecin^30^, paclitaxel^31^, pirfenidone^32^ and adriamycin^30,33^. Since both GO and ZOL contain aromatic rings in their chemical structure, ZOL could conjugate with GO by non-covalent *π* − *π* interactions. Additionally, hydrogen bonding and hydrophobic interactions can also occur based on the ZOL and GO structures. Conjugation of ZOL on GO may increase the size of the complex reducing the fast renal filtration, and consequently, increasing the circulation time throughout the body^12^. Furthermore, the slow release of ZOL from GO may prevent harmful side effects which mostly result from the presence of high doses of the drug in the body^34^.

In this study, ZOL and GO were conjugated to increase the effect of ZOL on MCF-7 breast cancer cells and to elevate the mineralization of BM-MSCs. To this end, conjugated ZOL-GO complexes were prepared with different concentrations, and characterized by Alamar blue viability assay, Acridine orange staining, SEM morphological analysis and alizarin red staining. The results suggested that the conjugation of ZOL and GO did not affect the viability of MCF-7 breast cancer cells and BM-MSCs compared to pure ZOL. Despite this, the prepared complex significantly increased the degree of mineralization of BM-MSCs.

## Results

### Photonic characterization of the ZOL-GO complex

**Figure 1:**
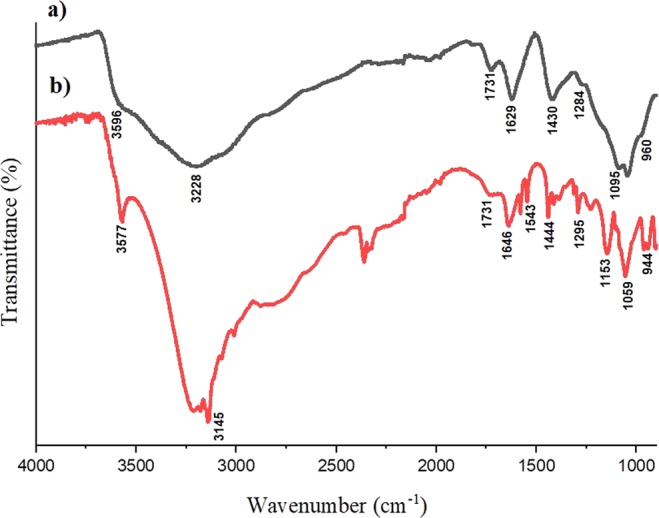
FTIR spectra of (**a**) GO, and (**b**) 200 µM −11.7 ng/ml ZOL-GO.

The FTIR spectra of GO and 200µM-11.7 ng/ml of ZOL-GO complex produced in this study are shown in Fig. 1.

In the FTIR spectrum of GO, the band at 3596, 3228, 1629 and 1284 *cm*^*−*1^ characterize the stretching vibration mode of O-H^35,36^. The band at 1731 *cm*^*−*1^ could be related to the C=O stretching vibration from the carboxyl group^35–38^. The O-H deformation vibration band was found at 1430 *cm*^*−1*^^38,39^.The band at 1095 *cm*^*−1*^ possibly arose due to C-O functional group^36^. Moreover, the band at 960 *cm*^*−*1^ can be ascribed to the epoxy groups in the GO structure^37,38^.

In the ZOL-GO spectrum, likewise to the case of GO, the bands at 3577 and 3145 *cm*^*−*1^ were related to the stretching vibration mode of O-H groups^35,36,40^. Additionally, bands at 1543 and 1646 *cm*^*−*1^ are attributed to the vibration of CH=CH groups in the imidazole rings; and the band at 944 *cm*^*−*1^ to the stretching vibration of C-C bonds^40,41^. The band at 1444 *cm*^*−1*^ can be attributed to stretching vibrations of C-H bonds in the imidazole ring^40–42^. The bands found at 1153 and 1295 *cm*^*−1*^ possibly arise due to P-O and P=O stretching vibrations, respectively^41^.

In the spectrum of ZOL-GO, in addition to the bands found for ZOL, there were two bands related to GO. The band at 1731 *cm*^*−*1^ was possibly due to the C=O stretching vibration from carboxyl groups of GO^35–38^. The band found at 1059 *cm*^*−1*^ possibly emerges due to the C-OH functional group of GO. The presence of these bands were proving conjugation of ZOLand GO.

### The cell viability and morphology of MCF-7 breast cancer cells

**Figure 2:**
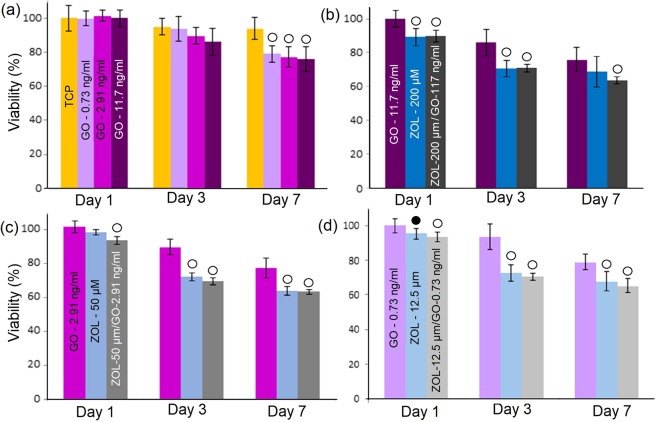
Percentage viability of MCF-7 cells after treatment with **(a)** different GO concentrations, and **(b-d)** selected concentrations of GO, ZOL and ZOL-GO. Metabolic activity was normalized to day 1 of TCP. The significant differences between TCP and other study groups is indicated by ^•^(P < 0.01 vs. control) and ^о^ (P < 0.05).

Figure 2 shows the viability of MCF-7 cells after GO, ZOL and GO-ZOL treatments.

According to Fig. 2, MCF-7 cells had a significant viability decrease after the treatment with all ZOL and ZOL-GO complexes on day 3. Incorporation of pure GO also significantly effected the percentage viability of MCF-7 cells. The percentage viability of MCF-7 cells decreased to 76% after addition of 11.7 ng/ml GO on day 7. After the treatment with 50 µM and 200 µM ZOL the percentage viability of MCF-7 cells dropped by 35%. Conjugation of GO and ZOL did not alter the %-viability of MCF-7 cells compared to ZOL.

**Figure 3:**
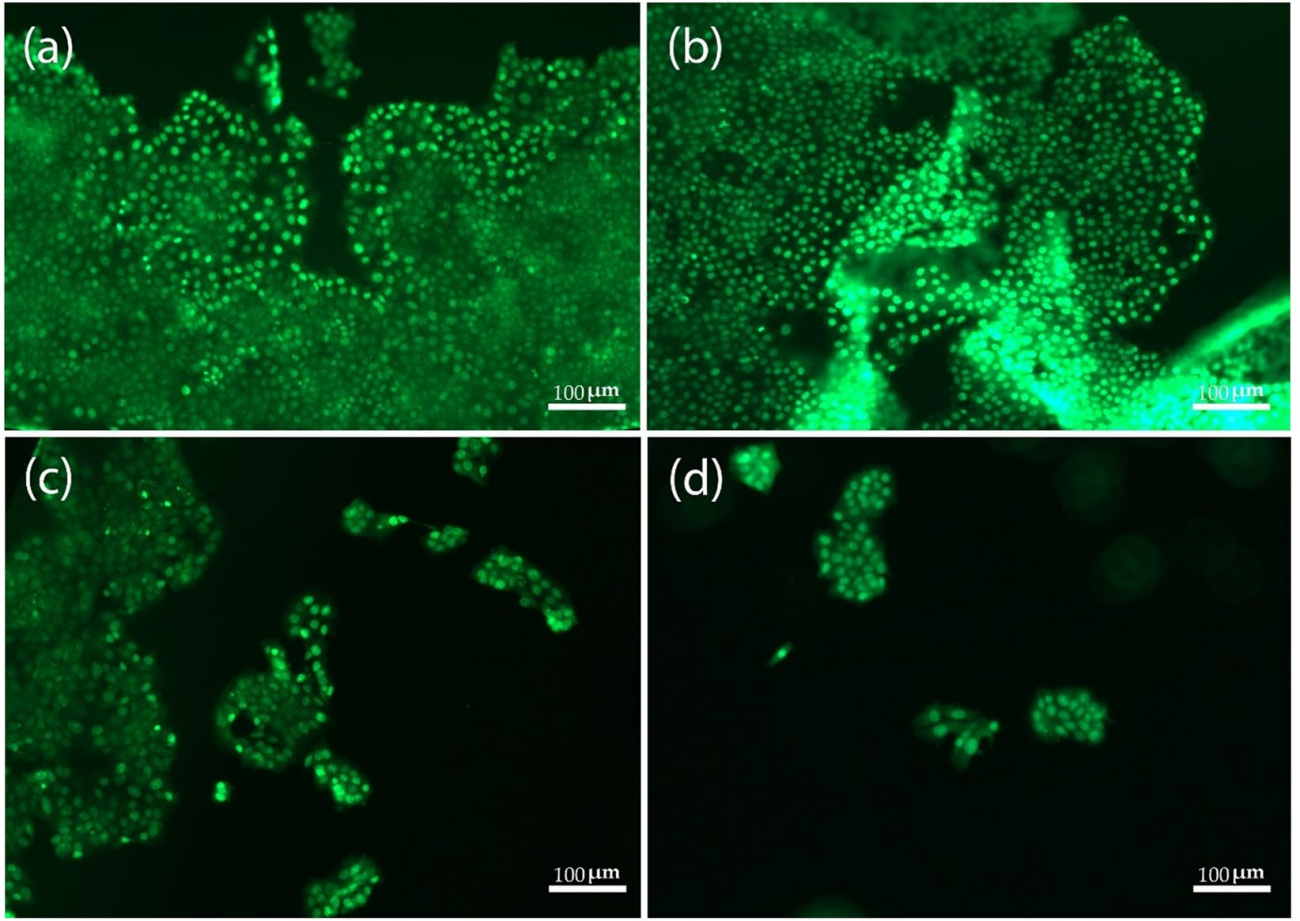
MCF-7 cells staining by AO. **(a)** TCP, and the samples treated with **(b)** 2.91 ng/ml of GO, **(c)** 50 *µ*M ZOL and **(d)** 50 *µ*M ZOL −2.91 ng/ml GO, after 3 days.

Figure 3 clearly depicts the viability of MCF-7 cells after the different treatments.

According to Fig. 3, the viability of MCF-7 cells was 83% and 87% for TCP and 2.91 ng/ml of GO sample, respectively. With the addition of ZOL, a viability of 34% was observed. Moreover, the cell viability was significantly lower after the ZOL-GO treatment which was only 9%. For the alamar blue assay, this drop was not as significant; however, the AO assay showed a more defined drop after the ZOL-GO treatment in comparison to the ZOL treatment. Table 1 shows percentage of live cells for different study groups.

**Table 1:** Composition of different ZOL-GO samples prepared.

GO-ZOL Samples	GO concentration (ng/ml)	ZOL concentration (µM)
1	11.7	200
2	2.91	50
3	0.73	12.5

**Figure 4:**
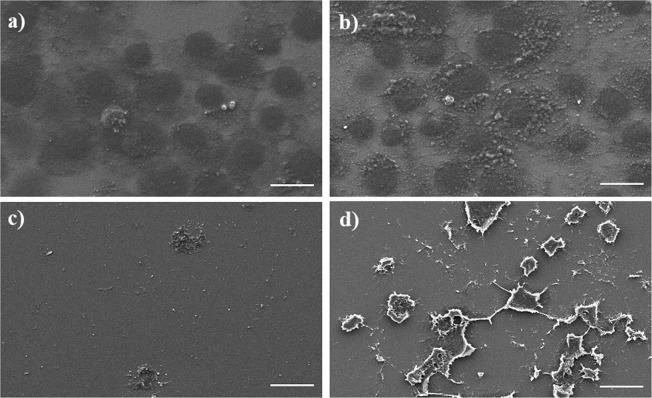
SEM images of MCF-7 cells after treatment using **(a)** TCP **(b)** 0.73 ng/ml GO **(c)** 12.5 µM ZOL **(d)** 12.5µM-0.73 ng/ml ZOL-GO (scale bar: 20 µm).

Figure 4 shows SEM images of MCF-7 cells after treatment using ZOL and GO for 3 days.

The MCF-7 cells had similar morphology and cell population features after the GO treatment and on TCP. The ZOL treatment led to a decrease in the number of cells.

### The cell viability of BM-MSCs

**Figure 5:**
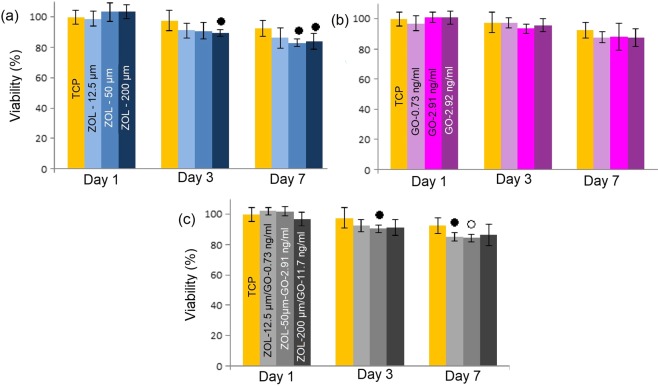
Percentage viability of MSC cells after treatment with different concentrations of **(a)** ZOL **(b)** GO and **(c)** ZOL-GO complexes (Metabolic activity was normalized to day 1 of TCP.) The significant differences between TCP and other study groups is indicated by •(P < 0.01 vs. control) and ^о^ (P < 0.05).

Figure 5 shows the viability of BM-MSCs after interaction with GO, ZOL and ZOL-GO complexes.

For BM-MSCs, pure GO did not significantly affect the viability of BM-MSCs with any of the concentrations used. ZOL did not cause a significant drop in the percentage cell viability on day 1. On day 3, there was only a considerable decrease for the concentration of 200 µM. On day 7, considerable decreases were found for both 50 and 200 µM ZOL. However, even on these days, the drop was only 8% larger than TCP. On the other hand, for ZOL-GO samples, on day 7, the most significant reduction of cells was observed for the 50 µM − 2.91 ng/ml ZOL-GO sample. This decrease was nevertheless still only 14% larger than TCP. The percentage of cell viability was over 83% for all study groups.

**Figure 6:**
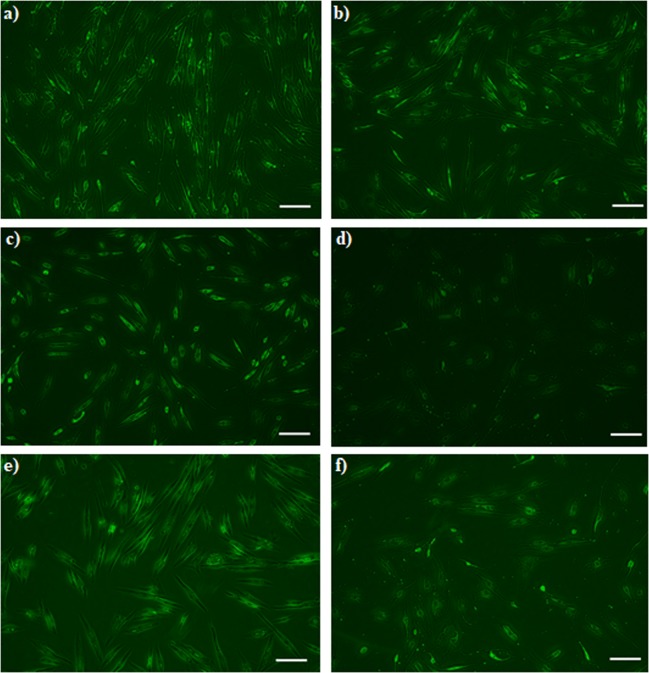
BM-MSCs staining by AO **(a)** 0.73 ng/ml GO **(b)** 11.7 ng/ml GO **(c)** 12.5 µM ZOL **(d)** 200 µM ZOL **(e)** 12.5 µM ZOL-GO **(f)** 200 µM ZOL-GO after 3 days (scale bar: 200 µm).

Figure 6 shows the viability of BM-MSCs after different treatments. Compared to TCP, with the addition of 0.73 ng/ml GO, the amount of BM-MSCs has increased, whilst the addition of 11.7 ng/ml GO, caused a slight decrease in the number of cells. With the addition of 12.5 µM ZOL, no significant decrease of the cell content was observed. However, an increase of the ZOL concentration to 200 µM significantly decreased the cell concentration, which was equivalent to the results obtained by the alamar blue assay (Fig. 5). For the cells treated with 12.5µM-0.73 ng/ml ZOL-GO, there was no decrease in the cell concentration compared to TCP. This was also paralleled by the results obtained by the alamar blue assay.

Additionally, in this case, the cells appeared to become more elongated, in comparison with the case observed in pure 0.73 ng/ml GO or 12.5 µM ZOL. For the cells treated with 200µM-11.7 ng/ml ZOL-GO, their MSC concentration was decreased. Despite this decrease, the cell concentration was much higher in comparison to the case of 200 µM ZOL. Also, compared to 11.7 ng/ml GO, the cells concentration was not significantly decreased, confirming that GO had a positive impact on the viability of BM-MSCs when complexed with ZOL.

### The cell morphology and mineralization of BM-MSCs

**Figure 7:**
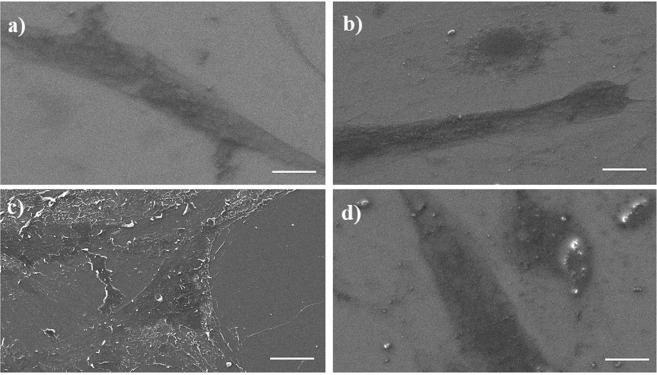
SEM images of BM-MSCs after treatment using **(a)** TCP, **(b)** 0.73 ng/ml GO, **(c)** 12.5 µM ZOL **(d)** 12.5 µM ZOL -0.73 ng/ml GO (scale bar: 20 µm).

Figure 7 shows images of an SEM study of BM-MSCs after treatment using ZOL, GO and ZOL-GO for 3 days.

**Figure 8:**
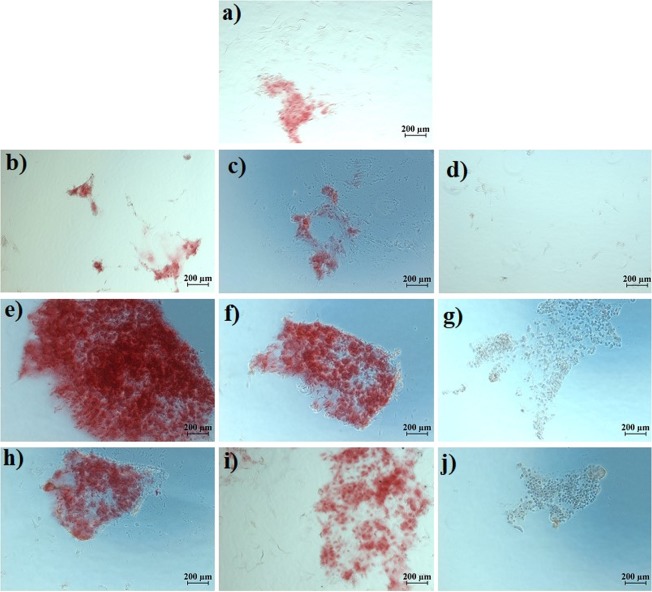
Alizarin red results of (**a)** TCP, (**b)** 0.73 ng/ml GO, (**c)** 2.91 ng/ml GO, (**d)** 11.7 ng/ml GO, (**e)** 12.5 µM ZOL, (**f)** 50 µM ZOL, (**g)** 200 µM ZOL, (**h)** 12.5 µM ZOL -0.73 ng/ml GO (**i)**, 50 µM ZOL - 2.91 ng/ml GO, and (**j)** 200 µM ZOL -11.7 ng/ml GO, on the day 14.

According to the images, BM-MSCs had a similar morphology after treatment with GO, ZOL-GO and TCP. Figure 8 shows the alizarin red results obtained on day 14.

**Figure 9:**
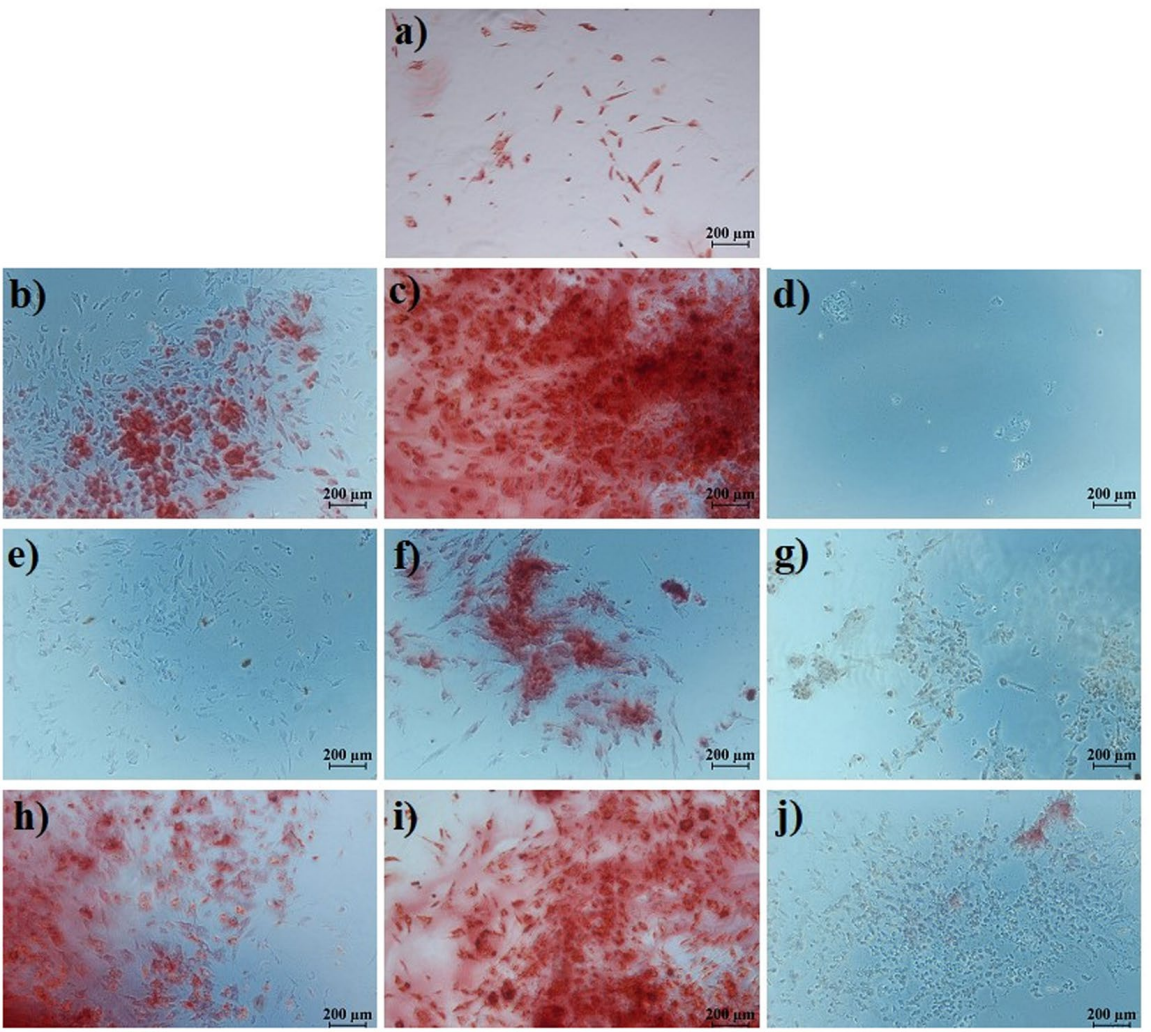
Alizarin red results (**a)** TCP, (**b)** 0.73 ng/ml GO, (**c)** 2.91 ng/ml GO, (**d)** 11.7 ng/ml GO, (**e)** 12.5 µM ZOL, (**f)** 50 µM ZOL, (**g)** 200 µM ZOL, (**h)** 12.5 µM ZOL- 0.73 ng/ml GO, (**i)** 50 µM ZOL -2.91 ng/ml GO, (**j)** 200 µM ZOL -11.7 ng/ml GO, on the day 21.

In general, on day 14, there was not much mineralization on the TCP and the GO treated samples. On the other hand, 12.5 µM ZOL led to a significant amount of mineralization. The degree of mineralization disappeared with the increase of ZOL concentration to 200 µM. ZOL-GO with the lowest concentration also caused mineralization; however, samples treated with 200 µM -11.7 ng/ml ZOL-GO showed no sign of mineralization. Figure 9 shows the alizarin red results on day 21.

On day 21, GO treated samples with a concentration of 0.73 and 2.91 mg/ml exhibited mineralization; however, no mineralization was observed for 11.7 ng/ml GO. For ZOL samples, there was a significant degree of mineralization for 50 µM ZOL, but no mineralization was observed for lower or higher concentrations. For ZOL-GO, there was a much more significant degree of mineralization than for pure ZOL for concentrations of 12.5 µM and 50 µM ZOL-GO. However, 200 µM ZOL-GO treated sample showed a minimal degree of mineralization. According to the alizarin red results, 50 µM ZOL significantly increased the mineralization of the cells.

## Discussion

In this study ZOL-GO complexes were produced for potential drug delivery purposes. FTIR results indicated that the drug complexes were produced successfully. These observations were supporting our results from a preliminary study where we have proven conjugation of ZOL and GO by using UV-vis spectroscopy^43^.

Alamar blue result was in accordance to the results obtained by other groups which indicated that ZOL inhibits MCF-7 cell proliferation and invasion at a dose level of 50 µM^44^. In the literature, the inhibition of breast cancer cells by ZOL was related to inhibition of farnesyl pyrophosphate (FPP) synthase in the mevalonate biosynthetic pathway^13,45–47^. Firstly, the inhibition of FPP synthase prevent prenylation of GTP-binding proteins such as Rho, Rac Ras and Rab due to depletion of farnesylpyrophosphate (FPP) and geranylgeranylphyrophosphate (GGPP)^45,48–50^. The prenylation of GTP-binding proteins are essential for cell survival and their impairment leads to cell apoptosis^51^. Secondly, FPP synthase inhibition leads to accumulation of isopentenyl pyrophosphate (IPP) and apoptotic ATP analogue (Apppl)^52^. Apppl inhibit adenine nucleotide translocase in the mitochondria and IPP activates Vγ9Vδ2-T cells which eliminate cancer cells^45^. The synergistic effect of ZOL and GO on the mevalonate pathway remains unknown and requires further research.

% cell viability observed via acridine orange assay shows that ZOL-GO provides a synergistic effect, reducing the % viability of MCF-7 significantly more than if solely ZOL was used. This is possibly due to improvement of the drug efficiency in the presence of GO carrier. GO leads to the effective internalization of the drug to the cells by endocytosis and/or cell penetration through lipid bilayer^53^. Utilization of the ZOL-GO complex led to a morphological change in MCF-7 cells, which requires further investigations.

Alamar blue study on BM-MSCs showed that ZOL-GO complexes did not have a significantly adverse effect on BM-MSC. These results align with a number of other studies which also suggests that ZOL does not have a negative effect on BM-MSC viability^54–57^. Overall, ZOL and ZOL-GO complexes did not drastically decrease the viability of BM-MSCs, but more significantly that of MCF-7 cells, which is advantageous for bone metastasis treatment.

Moreover, acridine orange assay results indicated that ZOL-GO complex led to elongation of BM-MSCs. In the literature, the elongation of MSCs has been related to the differentiation of MSCs to osteoblasts^58–60^. In our study, the combination of GO and ZOL could possibly trigger the differentiation of cells to osteoblasts. Literature also suggests that both GO and ZOL are effective at differentiating MSCs to osteoblasts at certain concentrations^61–63^. At high concentrations, ZOL was found toxic to cells which led to decrease of % viability of BM-MSCs^64,65^.

Additionally, SEM results revealed that the samples treated with ZOL-GO shows the presence of clusters which might be due to the indicated mineralization of BM-MSCs. The morphology of ZOL treated samples exhibits a change, compared to the other samples, which could be due to mineralization of the cells under the influence of the ZOL treatment, which is in agreement with reports in the literature^57,66^. Ebert *et al*.^57^, Carbonare *et al*.^62^ and Hu *et al*.^67^ also indicated that at the µM level, bisphosphonates trigger the osteogenic differentiation of MSCs, leading to their mineralization. Additionally, GO also leads to mineralization of MSCs, therefore in this study we have observed a synergistic effect of ZOL-GO complexes on mineralization of MSCs^61–63^.

According to the studies of Igarashi *et al*.^68^ and Pan *et al*.^69^, the addition of 25 µM ZOL increased the mineralization of cells. However, lower concentrations did not lead to a considerable degree of mineralization. Accordingly, Basso *et al*.^70^ also found inhibition of mineralization with application of only 5 µM of ZOL on osteoblast cells. Vaisman *et al*.^71^ and Patntirapong *et al*.^11^ indicated that higher doses of ZOL than 100 µM inhibited the mineralization of bone^66^. This is also in agreement with the results of the current study. Gao *et al*.^72^ indicated that ZOL induces osteogenic differentiation and mineralization via inhibition of mammalian target of the rapamycin complex 1 (mTORC1) activity. This leads to an upregulation of the bone morphogenetic protein-2 (BMP-2) and osteocalcin (OCN) gene expressions^69,72^. At higher doses, differentiation and mineralization were prevented via promotion of the mTORC1 activity^72^.

As indicated earlier, GO induces mineralization of cells^28,73–75^. Therefore, in our case, the ZOL-GO complex exhibited a synergistic effect, improving the mineralization of cells to high degrees, greater than those obtained solely by GO or ZOL. The results obtained are interesting and provide an opportunity to enhance the efficiency of the ZOL drug by using carbon-based carriers. Future research should concern *in vivo* studies to evaluate the performance of ZOL-GO complex structures in more details. Animal models may be used after conducting an appropriate harm-benefit analysis, considering animal rights and ethical issues beforehand. Moreover, anti-cancer effects should be studied correlating interactions with gene expressions, such as Bax and Bcl-2.

## Conclusion

In this study, different concentrations of GO were loaded on ZOL, and the effects of ZOL, GO and ZOL-GO complexes on the characteristics of the BM-MSCs and MCF-7 cell lines were studied. ZOL did not significantly reduce the viability of BM-MSCs, and GO had no effect on the viability of BM-MSCs. On the other hand, both ZOL and ZOL-GO complexes significantly decreased the viability of MCF-7 cells. Staining of cells led to results that were equivalent to the alamar blue study. Alizarin red results showed that ZOL with a concentration of 50 µM induces a high degree of mineralization. This was significantly increased with the addition of GO to 50 µM ZOL. Overall, the results revealed that ZOL-GO complexes could decrease the viability of MCF-7 breast cancer cells, whilst they did not drastically affect the viability of BM-MSCs. Additionally, 50 µM-2.91 ng/ml ZOL-GO caused a significant degree of BM-MSCs mineralization. Such ZOL-GO complexes show a promising performance for the drug treatment of osteoporosis and metastasis.

## Experimental

### Preparation of ZOL, GO and complexes

To prepare ZOL samples, 5 ml of ultra-pure water was added to 10 mg of zoledronic acid monohydrate (Sigma-Aldrich, >98%, HPLC) to obtain the stock solution with a concentration of 2 mg/ml. Then, three different samples with concentrations of 12.5, 50 and 200 µM were obtained by diluting the stock solution with DMEM (Dulbecco’s Modified Eagle Medium), and magnetically stirring (Isolab-340) over night. To prepare various concentrations of GO, 4.5 ml of ultra-pure water was added to 0.5 ml of GO (Sigma- Aldrich, 2 mg/ml, mean sheet diameter: 22 µm) to form the stock solution which was stirred by a vortex at room temperature for 30 min. The stock solution was then diluted with DMEM and stirred for 30 minutes by a sonicator to obtain solutions with GO amounts of 11.7, 2.91 and 0.73 ng/ml.

Finally, ZOL-GO complexes with different compositions were prepared by mixing of the GO and ZOL suspensions, as shown in Table 2. To dilute GO and ZOL suspensions in order to achieve the required concentrations, the stock solutions were diluted with DMEM, stirred by the sonicator for 15 min, and then magnetically stirred overnight.

**Table 2:** Percentage of live MCF-7 cells for different study groups.

Sample groups	% of live cells
TCP	83
GO	87
ZOL	34
ZOL-GO	9

### Characterization methods

FTIR spectroscopy was performed by using Nicolet FTIR Instruments, Thermofischer to analyze the chemical structure of ZOL conjugation with GO at the wavenumber regime from 4000 to 400 cm ^−1^ with a 2 cm^−1^ resolution^76^.

To study the performance of ZOL-GO drug complexes, cell culture studies were carried out using the human breast cancer cell line (MCF-7, ATCC HTB-22) and human bone marrow-derived MSCs (BM-MSCs, ATCC PCS-500-012).

For the alamar Blue assay, the cell line of MCF-7 cells were plated in a 96 well-plate (Sigma) with a density of 5 × 10^3^ cell/cm^2,77^ and BM-MSC with a density of 2 × 10^3^ cell/cm^2^ in complete growth medium with DMEM (Sigma), 10% Fetal Bovine Serum (FBS) (Sigma) and 1% penicillin/streptomycin (Sigma) at 37 °C with 5% CO_2_^67^. For BM-MSC, additionally, 4 mM L-Glutamine (Sigma) was added into the growth medium. 24 hours after seeding of the cells, they were treated with samples including ZOL, GO and ZOL-GO complexes in total growth medium. The drug treatment in this assay was applied on day 1, 3, and 7. For the alamar blue assay, 10% of alamar blue solution (Sigma) was mixed with DMEM and 100 µl of the prepared solution was added in each well on day 1, 3, and 7^78^. A microplate reader (BIO-RAD Mark, Microplate Reader) was used to study viability of the cells^79^. Statistical analyses were conducted by one-way analysis of variance (ANOVA) with post-hoc Tukey test.

Both BM-MSC and MCF-7 cells were seeded in a 48 well-plate at a density of 7.5 × 10^3^ cells per well and were incubated with the complete medium overnight. Then, cells were treated with the samples for 3 days. The wells were washed with the media twice and Acridine orange (AO) was added to the wells to stain the live cells. Images were taken after a few seconds by inverted phase contrast microscopy. Open-source software ImageJ was used to calculate the percentage of live MCF-7 cells for AO staining.

A FEI-Philips XL30 Environmental Scanning Electron Microscope (SEM) with Field Emission Gun, equipped with an Energy Dispersive X-ray (EDX) analyzer, was used operating at 5 kV in the secondary electron imaging mode for the study of morphological changes of BM-MSC and MCF-7 cells after the drug treatment on day 3. For the investigations, 2.5% glutaraldehyde was added on the cells for the fixation purpose in a dark room. For dehydration, the samples were immersed in 30%, 50%, 80%, and 95% ethanol for 2 minutes each. Then, the ethanol was removed and a few droplets of hexamethyldisilazan (HMDS) were added. The samples were left to become dry overnight^80^.

For alizarin red staining, BM-MSC with a density of 10 × 10^3^ cell/cm^2^ was seeded in a 24 well-plate and treated with the growth medium. After 24 hours, the cells were treated with osteogenic differentiation medium, comprising of the complete growth medium, 50 µg/ml of L-Ascorbic acid-2-phosphate, 5 mM of β-Glycerophosphate and 10 nM of Dexamethasone; all purchased from Sigma, to achieve final concentrations as shown in Table 1. The osteogenic differentiation medium was changed every 2 or 3 days. Alizarin red salt was used for the staining of free calcium ions and certain calcium compounds as an indicator of the mineralization. First, cells were fixed with 4% paraformaldehyde and 3xDPBS for 30 minutes at 4 °C and then washed with Dulbecco’s phosphate-buffered saline (DPBS) twice^81^. Thereafter, 2% alizarin Red S was added and the suspension was kept for 45 minutes in the dark at room temperature. The solution was removed and cells were washed with DPBS. Then, cells were observed by fluorescence microscopy (Axio Vert A.1-Zeiss) on days 14 and 21.
